# Reindeer habitat use in relation to two small wind farms, during preconstruction, construction, and operation

**DOI:** 10.1002/ece3.2941

**Published:** 2017-04-21

**Authors:** Anna Skarin, Moudud Alam

**Affiliations:** ^1^ Department of Animal Nutrition and ManagementSwedish University of Agricultural SciencesUppsalaSweden; ^2^ Section of StatisticsSchool of Technology and Business StudiesDalarna UniversityFalunSweden

**Keywords:** anthropogenic disturbance, before after design, pellet group count, *Rangifer*, renewable energy development, Sámi reindeer husbandry, spatial correlation

## Abstract

Worldwide there is a rush toward wind power development and its associated infrastructure. In Fennoscandia, large‐scale wind farms comprising several hundred windmills are currently built in important grazing ranges used for Sámi reindeer husbandry. In this study, reindeer habitat use was assessed using reindeer fecal pellet group counts in relation to two relatively small wind farms, with 8 and 10 turbines, respectively. In 2009, 1,315 15‐m^2^ plots were established and pellet groups were counted and cleaned from the plots. This was repeated once a year in May, during preconstruction, construction, and operation of the wind farms, covering 6 years (2009–2014) of reindeer habitat use in the area. We modeled the presence/absence of any pellets in a plot at both the local (wind farm site) and regional (reindeer calving to autumn range) scale with a hierarchical logistic regression, where spatial correlation was accounted for via random effects, using vegetation type, and the interaction between distance to wind turbine and time period as predictor variables. Our results revealed an absolute reduction in pellet groups by 66% and 86% around each wind farm, respectively, at local scale and by 61% at regional scale during the operation phase compared to the preconstruction phase. At the regional, scale habitat use declined close to the turbines in the same comparison. However, at the local scale, we observed increased habitat use close to the wind turbines at one of the wind farms during the operation phase. This may be explained by continued use of an important migration route close to the wind farm. The reduced use at the regional scale nevertheless suggests that there may be an overall avoidance of both wind farms during operation, but further studies of reindeer movement and behavior are needed to gain a better understanding of the mechanisms behind this suggested avoidance.

## 1 Introduction

The boreal forest in Fennoscandia represents an important grazing resource for reindeer (*Rangifer tarandus tarandus*) within the Sámi reindeer husbandry system in Northern Europe. The forest has been exposed to major changes due to forestry and other exploitation, such as mining and hydro power, over the last century (Herrmann et al., 2014; Sandström et al., 2016). More recently, there has been a massive increase in the planning and construction of large wind farms in the forest area, comprising several hundred windmills together with their associated infrastructure of roads and power lines. For example, in the four northern counties of Sweden, there are currently 902 wind turbines in place, another 1,588 are planned and applications have been submitted for a further 2,143 (www.vindbrukskollen.se, December 2016), with little knowledge of the possible adverse effects on reindeer husbandry and the habitat use and migration of free‐ranging reindeer, as well as other species.

Wind turbines usually have a running phase of 20–25 years. An increased knowledge of the impacts on reindeer habitat use is critical in order to mitigate those impacts from work already in the planning phase (Northrup & Wittemyer, 2013). In addition, disturbance associated with the human activity within the wind farm, as well as noise from wind turbines, might disturb animals, hindering vocal communication and their ability to hear predators (Biedenweg, Parsons, Fleming, & Blumstein, 2011; Rabin, Coss, & Owings, 2006). Furthermore, prey animals like reindeer react to movements in their sight (Favreau, Goldizen, & Pays, 2010; Heesy, 2004) and may, therefore, react to the movement of the turbine blades. To date, three studies have examined the effects of wind farm construction and operation in relation to reindeer habitat use and these found limited to large effects of the wind farms (Colman et al., 2012, 2013; Skarin et al., 2015). Studies of reindeer habitat selection in relation to wind farm development from the boreal forest landscape are scarce (cf. Skarin et al., 2015).

Reindeer are opportunistic feeders constantly moving over large areas while feeding, following the vegetation phenology during the snow‐free season to exploit early stages of plant growth (e.g., Iversen et al., 2014; Skarin, Danell, Bergstrom, & Moen, 2010). The free‐ranging reindeer associated with Sámi reindeer husbandry in Fennoscandia roam freely for most of the year, especially during the snow‐free season when the herders only gather the reindeer for calf marking in the middle of the summer (Skarin et al., 2010). In the winter and during migrations between summer and winter ranges, they may be more constrained in their movements by herders’ every‐day actions.

Studying habitat selection by herbivores, such as reindeer, demands a hierarchical or multiscale approach to minimize the risk of missing important behavioral responses to factors that have a different impact at different scales (Northrup, Anderson, Hooten, & Wittemyer, 2016; Senft et al., 1987; Skarin & Åhman, 2014). Noninteractive factors, for example, barriers, have an impact on habitat selection at the large geographical scale and may limit selection at local scales (Northrup et al., 2016; Senft et al., 1987). Earlier studies of reindeer and caribou show clear patterns in avoiding infrastructure and human activity over relatively large distances, sometimes several kilometers (e.g., Beyer et al., 2016; Johnson, Ehlers, & Seip, 2015; Skarin & Åhman, 2014; Vistnes & Nellemann, 2008). Vistnes and Nellemann (2008) found that studies examining the effect of disturbance on reindeer and caribou within 2 km of the source and/or during short time periods often did not reveal any effect of disturbance. The majority of the studies in which the effects over distances greater than 2 km and/or during a longer time period were considered, revealed negative effects of the disturbance on the reindeer (Vistnes & Nellemann, 2008). This means that regional and long‐term studies are required to safeguard against underestimating the most important effects and to capture how the free ranging herded reindeer react to changes in the landscape. Nonetheless, a drawback when increasing the scale of a study is the problem of confounding effects among the landscape metrics, which can potentially enhance or reduce the effect of a disturbance (Clevenger & Waltho, 2005). Therefore, it is necessary to select the explanatory variables carefully and thus reduce correlations between them, or to evaluate ways to remove correlations before interpreting the results of any study (Graham, 2003).

A common and often recommend tool to evaluate environmental impact and ways to remove distortions due to correlations is the so‐called Before‐After‐Control‐Impact (BACI) experimental design (Kuvlesky et al., 2007; Strickland et al., 2011). In such a design, two parallel study areas are evaluated before and after development: One where the development takes place and another similar area that serves as a control. However, two calving areas, for example, might be difficult to compare at the regional scale because of large variations in landscape conditions (including other infrastructure) between different reindeer herding communities. An alternative to BACI design is to perform a before after (BA) study over an area large enough to capture the effects at the regional scale, and at the same time try to control for the differences between years that do not depend on the development.

Pellet group counts, as a technique to survey an animal's habitat use, have the advantage that the overall animal abundance over several months is captured at a large spatial scale, based on a concentrated recording effort (Marques et al., 2001). A large number of habitat selection studies on large herbivores (including reindeer) have used fecal pellet group counts to collect data on habitat use (e.g., Guillet et al., 1995; Harkonen & Heikkila, 1999; Mansson, Hauser, Andren, & Possingham, 2011; Neff, 1968; Quayle & Kershaw, 1996; Skarin, 2007). However, these recent works on the analysis of habitat preferences failed to address the issue of spatial correlation among their pellet group count observations in their analysis. Failing to account for zero‐inflation and correlation can induce substantial bias in the estimates of effects (Kassahun et al., 2014) and invalidate any statistical inference, due to the violation of the mean−variance relationship imposed by ordinary generalized linear models, for example, binomial and Poisson's models (Kassahun et al., 2014; Lee et al., 2016). Using a suitable hierarchical generalized linear model (Lee & Nelder, 1996) with spatially correlated random effects, for example, a spatial Poisson (Lee et al., 2016) or a binomial model, we may be able to overcome this problem.

The aim of this study was to evaluate whether reindeer habitat use, measured by fecal pellet group abundance, in the vicinity of the wind farms was lower during the construction and operation phase of two relatively small wind farms (8 and 10 wind turbines, respectively) compared to the period before construction. Because the reindeer are likely to be annoyed by the sound and sight of the wind turbines as well as the increased human activities (e.g., sound from vehicles associated with wind farm construction and maintenance), we hypothesize that they would avoid or decrease their usage of the areas nearby the wind farm during the construction and operation phases compared to the preconstruction phase. This hypothesis was tested using a BA design (Strickland et al., 2011). Overall impact of the wind farm construction and operation phase on reindeer habitat use was evaluated at both regional (reindeer range used from calving to autumn, within 15 km of the wind farm) and local (wind farm mountains within 2 km from wind turbines) scales using pellet group counts in relation to distance to wind turbine in association with time period, after controlling for the possible confounding effects, and spatial correlation.

## 2 Material and Methods

### 2.1 Study area

**Figure 1:**
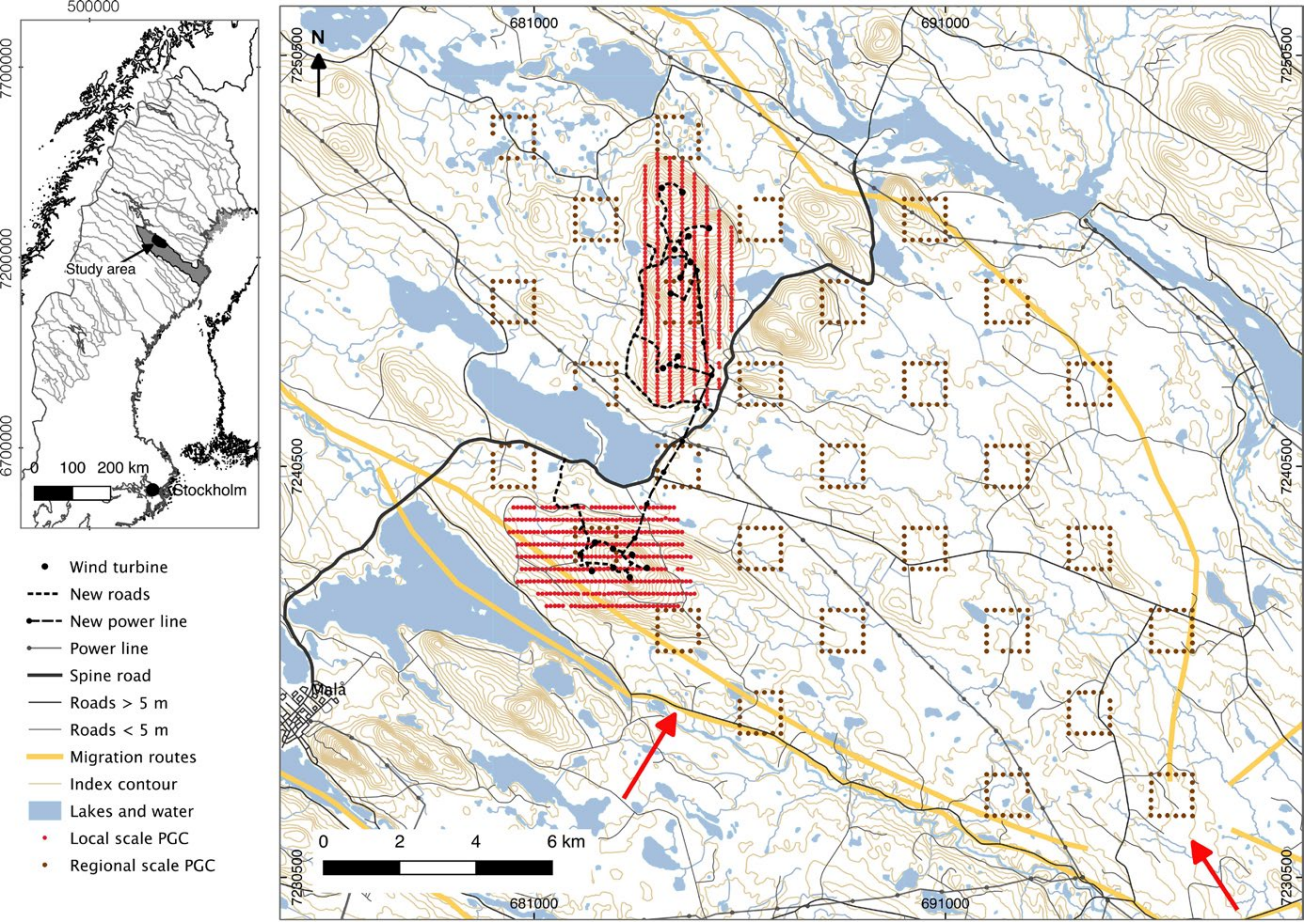
Map showing part of the calving area of the Malå reindeer herding community and the study area, with local scale pellet group count plots marked with red dots, the regional scale pellet group counts marked with brown dots, and the important reindeer migration routes in the herding community marked in yellow. Arrows show approximate sites where the reindeer were let out into the calving ranges after the migration “by foot” controlled by the herders ©Lantmäteriet i1204/764

The study was conducted in and around the two wind farms near to each other, on Storliden mountain and Jokkmokksliden mountain (where eight and 10 wind turbines, respectively, were constructed in 2010–2011), located in the calving and postcalving ranges of the Malå forest reindeer herding community (65°14′, 18°58′) in northern Sweden (Figure 1). The study area was used by a part of the total herd: approximately 1,200 female reindeer and their calves (the total number of female reindeer in the whole herd ranged between 4,144, and 4,854 over the study years). Every year in April, migration took place (“by foot” in all years except 2015, when the reindeer were moved with trucks), with the reindeer herd moving from the winter ranges in the east to the summer ranges in the west. After migration, the reindeer were released into the study area (see arrows in Figure 1) at the beginning of May (2 May 2009, 10 May 2010 and 2011, and 1 May 2015; for the other years, we do not have an exact date of arrival). The reindeer used the area primarily during the calving season and then moved in and out of the area during the whole snow‐free season until late autumn when they were moved back to the winter ranges. At the end of June, reindeer were gathered by the herders for calf marking and moved to the closest corral, thus redistributing the reindeer to some extent. In the early autumn (from the end of August), reindeer concentrated their activity toward the southern side of Storliden before they moved out of the area freely or because of the herders’ action.

The study area is characterized by a boreal forest landscape, interspersed with mires, lakes and hills or smaller mountains, with the forested land comprising old growth forest, clear cuts, and plantations. The whole study area is a managed forest; when the trees are <5 m, it is classified as young forest and open areas with trees <2 m are classified as clear cuts (Reese et al., 2003). According to the herders, the reindeer prefer Storliden just after calving. Apart from the forest felling for the wind farm construction, two harvesting operations took place in the study area during the study period: At Storliden two sections of old forest (22 and 41 ha, >120 and >70 years), 600 m north and 1200 m west of the wind farm were clear cut in May 2012 and in July 2013, respectively.

### 2.2 Wind farm construction site and existing infrastructure

The year 2009 was defined as the predevelopment year (hereafter referred to as the “pre‐construction” phase), during which the area had existing infrastructure such as a road network, power lines, and an underground mine on the north side of Storliden (this ceased working in 2008). Construction work started on 10 May 2010 at Jokkmokksliden, and on 1 June 2010 at Storliden. To access the wind farms, 22 km of roads was constructed, with 8.5 km of 36 kV power lines connecting to the existing power grid via the new utility station built in between the wind farms. The full infrastructure at Jokkmokksliden, was developed during 2010 and the first five wind turbines were constructed, with five more turbines being erected in 2011. At Storliden, the road network and power lines were established in 2010 and the turbines were put in place in 2011. All turbines started to generate electricity in November 2011. The years 2010 and 2011 are hereafter referred to as the “construction” phase, as development continued throughout. The years after construction (2012–2015) are here referred to as the “operation” phase, as the wind turbines were running.

### 2.3 Pellet group counts

**Figure 2:**
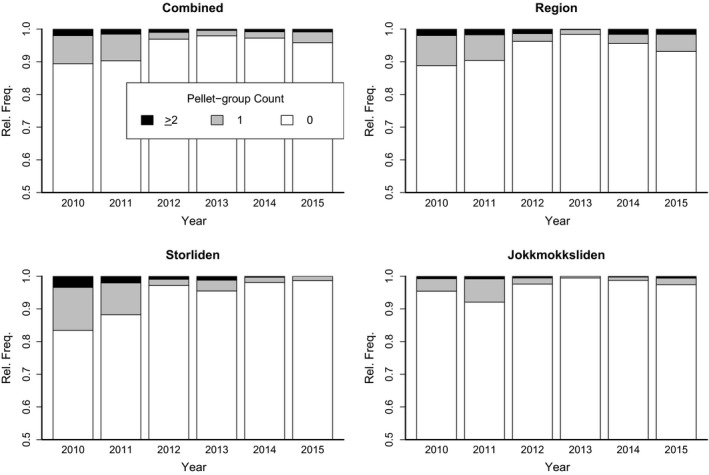
Bar graphs of the relative frequency of pellet group counts within each study year for all data combined, data from the count at the region scale, and data from the count at the local scale from Storliden and from Jokkmokksliden (2010 – Preconstruction, 2011 and 2012—Construction, and 2012–2014—Operation)

The fecal pellet group count, used as a proxy to measure habitat use by the reindeer, was conducted every year (2010–2015), over a period of 4–9 days between 24 May and 8 June (Table 1). The pellet groups were counted using the fecal accumulation rate in 2010–2015 (Campbell, Swanson, & Sales, 2004; Skarin, 2007). Thus, in 2009, the plots were positioned and marked with a wooden stick, and after the pellet groups had been counted, they were removed from the plots; in subsequent years, we counted the number of pellet groups in the plots once a year and then cleaned the plots. The count in 1 year mainly represented the reindeer use of the area in the previous summer and the calving period the same year, as we inventoried the plots as soon as the area was accessible after snow‐melt and before greening up to avoid pellet groups being hidden by understory vegetation, that is, at the end of the calving season each year. Thus, the inventory in 2010 mainly represented the reindeer use prior to the construction of the wind farm and the first 15 days of the construction work at Jokkmokksliden during calving and was treated as representative of reindeer use during the “preconstruction” phase. The inventory in 2011 solely represented the construction phase. The inventory in 2012 covered both part of the construction phase in 2011 and the operation phase in May 2012, and thus represented a mix of construction and operation phase (Table 1) plus in addition the forest activity at Storliden in May 2012. However, to keep things simple and to have a large enough sample to assure stability of the inference on the effects of the different phases, we treat the inventories from 2011 and 2012 as representative of the construction period. Because of the overlap of data collection in 2012 between operation and construction phase, conclusions drawn on the effect of construction period should be regarded as a mixed effect of construction and operation (with most weight being given to the construction period). The inventories from 2013, 2014, and 2015 represented the operation phase, except for the forestry activity in July 2013. It is generally believed that there is some random variation in habitat use by reindeer between years (Nicholson et al., 2016), which was also seen in the initial analysis of the data (Figure 2); therefore, it makes sense to average the usage over a number of years to get stable estimate of the effects of the wind farm.

**Table 1:** Date of pellet group counts in the Malå reindeer herding community, including phases for Storliden and Jokkmokksliden wind farm development and how this corresponded to reindeer use of the area (note that reindeer did not use the area during the winter seasons from November to April)

Phase of wind farm development	Date for pellet group count	Date of reindeer use represented by each count
Inventory not used in statistical analysis	1–9/6 2009	May in 2009 and 2008 and prior to this depending on pellet group decay rate within each vegetation type
Preconstruction and 15 days construction	28/5–1/6 2010	10/6 2009–27/5 2010
Construction	23/5–26/5 2011	2/6 2010–22/5 2011
Construction and 30 days operation	28/5–1/6 2012	27/5 2011–27/5 2012
Operation	27/5–2/6 2013	2/6 2012–26/5 2013
Operation	2/6–8/6 2014	3/6 2013–1/6 2014
Operation	25/5–29/5 2015	9/6 2014–24/5 2015

The study followed a point transect survey design (Buckland et al., 2001) at the local scale within 2 km of the wind farms, covering 15 km^2^ around each wind farm, and at the regional scale within 15 km of the wind farm, covering 250 km^2^, the core of the reindeer calving range (Figure 1). In 2009, 1,315 plots were inventoried, and in the following years, 1,162–1,248 plots were inventoried. Each plot was 15 m^2^ (radius = 2.18 m). At the local scale, the distance between transects was 300 m and the distance between each plot on the transects was 100 m. At the regional scale, the plots were placed along the sides of 29 1 × 1 km squares, with 20 plots every 200 m along the perimeter of each square (with some minor deviations due to water and roads). The squares were distributed across over the study area, with more squares toward the eastern side of the main road in the area, as this was the main calving range in this part of the reindeer herding community and we could not cover the range further to the west due to time constraints. The squares were separated by a minimum distance of 1,400 m. To be counted, the center of the pellet group had to be inside the plot. As an animal might move as it defecates, the pellets could spread over a large area. Therefore, a cluster of 20 or more pellets was defined as a pellet group. If the pellets were evenly spread over the plot, we counted the separate pellets. As reindeer pellet groups have been estimated to contain 127 (±7) separate pellets (Skarin, 2007), 20–146 pellets were counted as one pellet group, and 147–273 pellets as two pellet groups, and so on.

### 2.4 Habitat variables

We included habitat variables known or suspected to be important predictors of reindeer habitat use (i.e., pellet group abundance) in a spatial binomial model (Table 2) (Skarin et al., 2015). The habitat variables included were vegetation type, elevation, slope, minimum distance to large (>5 m) and small (<5 m) roads, new roads, power lines, and the wind turbines within the wind farm. Before computations, all variables were screened for collinearity by calculating a variance inflation factor (VIF), and we used VIF ≥ 3.0 as a threshold for removing a variable. The environmental parameters were first extracted using Arc GIS 9.3^™^ software (ESRI Inc., © 1999–2009). All the digitized geographical data were provided by Lantmäteriet (http://www.lantmateriet.se), which supplies national geographic and land information data in Sweden. We used the Swedish Landcover Map for habitat type description. There are 43 vegetation classes from the Swedish Landcover Map present in the area (SMD, Lantmäteriet 2004). We complemented the SMD layer, which originates from satellite images from the year 2000, with satellite data for each study year to include changes from old forest to clear cuts and from clear cuts to young forest. To avoid rare vegetation type classes, the 43 classes were merged into five classes: forest, young forest, clear cuts, mire, and other (Table 2). The class variables were resampled from the 25 m grid to a 50 m grid, where the most common class (if equal the class was randomized) from the 25‐m grid determined the new class of the 50‐m grid. We used a digital elevation model (DEM) layer that had a resolution of 50 m and a vertical accuracy of ±2 m. The ruggedness index (VRM) was calculated from the DEM layer as described by Sappington, Longshore, and Thompson (2007) with a 5 × 5 neighborhood. To capture between‐year variations in weather conditions, we considered average precipitation from May to October in the previous year, daily average humidity and daily average snow depth. All weather data were downloaded for the Adak meteorological station (Lat—65.383, Long—18.6201) 20 km northwest from the wind farms, collected by the Swedish Meteorological and Hydrological Institute (http://www.smhi.se/).

**Table 2:** Proportion or ranges, with median value in parentheses, of environmental parameters (50‐m resolution for geographical parameters) within the Malå study area. Weather records are from the Adak (Lat—65.383, Long—18.6201) meteorological station (www.smhi.se) and geographical parameters are from Lantmäteriet (www.lantmateriet.se). Total size of the study area is 250 km^2^, including lakes

Environmental parameters	Range or percentage in whole study area
Vegetation type classes	
Forest (coniferous, mixed and broadleaved forest >5 m stem length)	34.7
Clear cuts (<2 m stem length)	9.9
Young forest (<5 m stem length)	20.3
Mires	27.1
Other	7.9
Continuous variables	
Elevation	263–529 (390) m
Slope (degrees)	0–19 (3.6)
Ruggedness index (VRM)	0–0.027 (0.0001)
Main road (>5 m)	0–4,103 (1,050) m
Forest road (<5 m)	0–2,487 (403) m
Wind turbines	0–15,296 (5,305) m
Power lines	0–5,411 (2,799) m
Water	0–1,632 (250) m
Precipitation mean May‐Oct/year	47–93 (71) mm
Snow depth/year	265–409 (322) mm
Relative humidity mean/year^a^	80–82 (81)%

a Relative humidity records are from the Malå‐Brännan (Lat – 65.1522, Long – 18.5974) meteorological station, as this was not recorded at Adak.

### 2.5 Statistical analysis of the pellet group count

The pellet group counts were treated as a count variable and an initial analysis showed that, within each year, over 83% of the plots contained no pellets and only about 2% (at most) of the pellet counts were greater than one (Figure 2). Moreover, we know that any reindeer visiting a particular location are also more likely to visit a nearby location than one far away from their current position. We therefore needed to use a count data model that could both handle excessive zero counts and account for spatial dependence in the outcome variable. Initially, we tried using a hurdle model (e.g., Hoef & Jansen, 2007) by fitting an additional truncated Poisson's model for nonzero counts. However, because the nonzero counts were extremely scarce, it was not possible to draw any conclusion from the fitted truncated Poisson models. We could thus assume that only the presence and absence of pellets mattered (not the exact counts). Lee et al. (2016) proposed a spatial Poisson hierarchical generalized linear model (HGLM; Lee & Nelder, 1996) based on the fact that high spatial correlation can explain excess zeros. We therefore model this using a hierarchical logistic regression (or binomial) model (Model (1)) where spatial correlation is also accounted for, via random effects.

To assess the regional scale, we analyzed the data collected from the squares separately from the local scale. We also combined the local and the regional data (hereafter referred to as the “combined” data) to determine whether spatially denser data would enhance our results at the regional scale. To assess the impact at the local scale, we analyzed data from Storliden and Jokkmokksliden separately, as the two areas are situated too far apart to expect any spatial dependence. For each data set, we fitted logistic models:

(1) $$E\left(I\left(y_{i,t}=0|u_{i,t}\right)\right)=p_{it};\;\text{logit}\left(p_{i,t}\right)=X_{i,t}\beta +u_{i,t}$$

where *i* = 1, 2, …, *n*_*t*_ (number of inventories), *t* represents the periods (preconstruction, construction, and operation), *X*_*i*,*t*_ represents the row of the design matrix associated with the fixed effects, *β*, for i:th location and at t:th time period, *I* is an indicator function, and **u **= {*u*_*i*,*t*_} ~ *N*(0,τ_1_(**I‐**ρ **D**)^−1^) where **D** is a neighborhood matrix whose diagonal elements are all zeros and the (*i*,* j*):th off‐diagonal element is 1 if the centers of the inventories *i* and *j* are located within 350 m of each other and 0 otherwise, and τ and ρ are parameters. The random effects, **u**, account for the spatial correlation between the neighboring plots, for ρ ≠ 0. Model (1) was fitted in R (R Core Team, 2016) using the hglm (Alam, Rönnegård, & Shen, 2015) package. The fitted logistic regression model showed underdispersion for all four data sets (Table 3). Hence, a quasilikelihood‐based inference, which allows the dispersion parameter of the mean model to vary from the theoretical binomial dispersion parameter, is justified for this data set.

**Table 3:** Fitted final models for the probability of counting any pellet group in a plot (binary part of hurdle) for all data combined, the region, Storliden, and Jokkmokksliden, with standard errors in parentheses

Parameter in logit model	Combined data from all areas (*SE*)	Region (*SE*)	Storliden (*SE*)	Jokkmokksliden (*SE*)
Intercept	−1.01^a^ (0.29)	−1.15^a^ (0.67)	−3.01^a^ (0.67)	−0.51 (1.48)
Distance^1^	0.05^a^ (0.02)	0.01 (0.07)	0.48 (0.27)	<0.01 (0.43)
Phases				
Preconstruction	0.00 (‐)	0.00 (‐)	0.00 (‐)	
Construction	−0.16 (0.14)	−1.12 (0.33)	−0.28 (0.47)	1.25^a^ (0.51)
Operation	−2.23^a^ (0.16)	−2.68^a^ (0.36)	−0.74 (0.53)	−2.21^a^ (0.62)
Clear cut	1.65^a^ (0.16)	1.58^a^ (0.46)	0.85 (0.62)	0.09 (1.10)
Forest	0.38^a^ (0.13)	0.49 (0.31)	0.08 (0.56)	−1.34 (0.79)
Young	0.18 (0.13)	0.29 (0.36)	−0.22 (0.55)	−1.35 (0.77)
Mire	0.00 (−)	0.00 (−)	0.00 (−)	0.00 (−)
Precipitation (10^−1^ m)	−3.45^a^ (0.31)	−4.82^a^ (0.45)	−1.43^a^ (0.56)	−5.69^a^ (0.56)
Distance: Construction	−0.05 (0.03)	−0.05 (0.05)	−0.27 (0.16)	−0.26 (0.21)
Distance: Operation	0.09^a^ (0.03)	0.11^a^ (0.05)	−0.60^a^ (0.19)	0.14 (0.25)
Dispersion parameter of the mean model	0.33	0.28	0.33	0.16
τ_1_	1.96	5.83	2.77	5.78
ρ_1_	0.05	0.22	0.08	0.08
No. observations used	7,175	2,991	1,948	2,190

^a^Significance at 5% level. A “0” estimate with missing standard error (indicated by “‐”) represents the reference category, for categorical covariates. ^1^Distance is measured as the square root of distance (in 100 m).

For each data, region, the combined data, and the data from Storliden and Jokkmokksliden we fitted model (1) with slope, VRM, the nearest distance from large roads (>5 m), small roads (<5 m), power lines, and water, dummy variables for clear cuts, young forest, forest, mires, precipitation, and time period (preconstruction, construction, and operation), and distance (in 100 m) from the nearest wind farms interacting with time period. DEM and distance to new roads were not used as a predictor variable as VIF >3.0, we also had to exclude humidity and snow depth, as they showed almost no variation between the years 2010 and 2015. All distance measures were square root transformed so as not to risk observations that were far away from, for example, turbines having a disproportionately large influence on the derivation of the model. We can assume that the reindeer's perception of something far away is less important for their choice of area, which could lead to an overestimate of the relevance of the variable if not transformed. Thereafter, we used a backward deletion method (Olsson, 2002, pp. 25–26) to obtain the final model. We kept only those terms that were significant at the 10% level. However, a main‐effect term was not deleted unless all interaction terms involving it had been deleted. To check the sensitivity of our results due to overlapping definition of phases and yearly variation (not explained by the covariates included), we refitted the final logistic model by (1) treating 2010, 2011, and 2012 as independent years while the operation phase (2013–2015) was kept unchanged, and (2) adding a random year effect.

To understand the strengths of the statistical inferences that we draw from the data, we checked the power of the tests in relation to the model parameters using a Monte Carlo simulation (see Appendix 2 for details). The calculation of an exact statistical power of the test was not possible as there is no close form solution for calculating the power of the test of the parameters in hierarchical generalized linear models. For this reason, the Monte Carlo method is widely used for power calculation with hierarchical models (e.g., Gelman & Hill, 2007; Green & MacLeod, 2016).

## 3 Results

Initial analysis of the raw data showed that, in general, the absolute pellet group abundance decreased by 61% in the whole region, 86% at Storliden, and 66% at Jokkmokksliden, from the preconstruction phase to the operation phase. More precisely, the mean numbers of pellet groups over the whole region and locally around Storliden decreased during the construction phase compared to the preconstruction phase, while there was a mean increase at Jokkmokksliden (mean differences, preconstruction–construction, (standard errors in parentheses): 0.06 (0.03) in the region, 0.13 (0.04) at Storliden and −0.01 (0.02) at Jokkmokksliden. A declining trend in the mean number of pellet groups per plot was found for all data between the construction and the operation phases (mean differences (construction—operation): 0.03 (0.01) in the region, 0.07 (0.02) at Storliden and 0.04 (0.01) at Jokkmokksliden).

The covariates of the model fitted for the regional data were vegetation type, average precipitation (from May to October in the previous year), and distance to wind farms in interaction with time period, and all other covariates were removed as they were insignificant. To ensure the comparability of the results between areas, we present each model with all the parameters that were found to be significant in at least one of the four models (Table 3).

The results from the first sensitivity check (1) showed (figures not presented in the article) a large variation between the years 2011 and 2012 in relation to distance to wind farm for all the fitted models, except for the model using the combined data. The model with the combined data showed that the interaction effects for 2011 and 2012 were both insignificant, which was also the case for the interaction term for construction phase with distance in the main model (Table 3). This indicates the importance of having abundant data to draw stable inferences. We thus decided to keep the main model unchanged. The results from the second sensitivity check (2) did not show any substantial difference in the parameter estimates and their inferential statistics, compared to those presented in Table 3. However, the inferences from the second sensitivity analysis should be regarded with caution because we had only six random effects, which may not be sufficient to produce a stable estimate of the respective variance components (Gelman & Hill, 2007, Ch. 1.29).

### 3.1 Regional scale

The fitted model using the combined data and the fitted model using data from the region showed that there was no significant response to the distance to wind farm during the construction phase (as mentioned above); however, the abundance of pellet groups increased significantly with increasing distance to the wind farms within the operation phase compared to the preconstruction phase (Table 3). The odds of observing at least one pellet group had decreased significantly (by 9% in the combined data, 11% in the regional data) between the preconstruction phase and the operation phase, as we moved 100 m toward the turbines (while everything else remained unchanged). The significant decrease in pellet groups between the preconstruction phase and the operation phase in the plots close to the sites of the wind farms suggests that reindeer avoided the wind farms at the regional scale when the turbines were in operation. There was a significantly higher selection for clear cuts in both the combined and the regional data and a lower selection for forests, young forests, and mires. Precipitation has a significant negative effect, that is, higher precipitation is associated with lower odds of finding any pellet, and this finding holds at the local scale too.

### 3.2 Local scale

**Figure A1:**
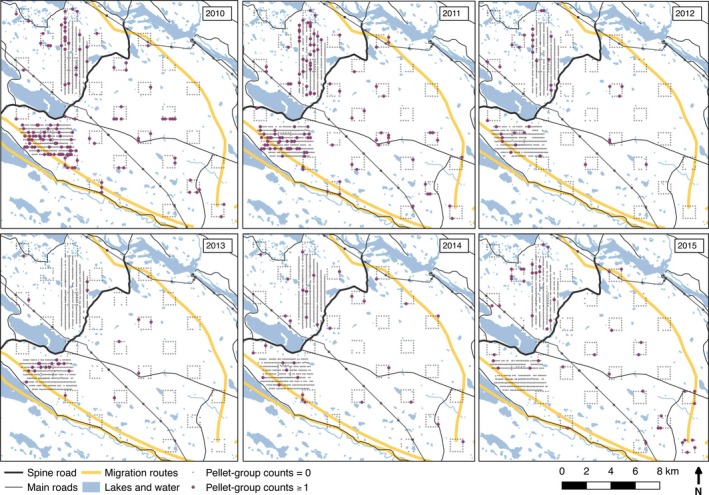
Maps of the spatial distribution of the results of the pellet group count separated by year from 2010–2015, around the Storliden and Jokkmokksliden wind farms within part of the calving, summer and autumn grazing range for the Malå reindeer herding community, Sweden. ©Lantmäteriet i1204/764

In Storliden, the odds of observing any pellet group in a plot increased significantly when moving toward the sites of the wind turbines, this trend was even stronger for the operation phase than when considering these locations during the preconstruction phase. This result suggests that the reindeer did not avoid the wind farm at the local scale around Storliden. Further, there was higher selection of clear cuts at Storliden. The density of the pellet groups at Storliden during the operation phase was concentrated toward the northern side of the mountain, in‐between the spine road and the wind farm (Figure A1).

The fitted model for Jokkmokksliden did not show any significant variation in the odds of observing a pellet group in a plot with increasing distance to the wind farm and time period. There was also limited use of areas with forest and young forest. In the Jokkmokliden area, the frequency of zero counts was already very high (compared to the data from Storliden and the whole region, see Figure 2). This indicates that Jokkmokksliden was never a favorite location for reindeer.

### 3.3 Simulation study

The results from the simulation study using the combined data, the regional data, and the Storliden data show that we generated an expected power not less than 0.51 for the interaction effect “sqrt (distance to wind turbine location in 100 m)*construction phase,” and not less than 0.63 for the other interaction term “sqrt (distance to wind turbine location in 100 m)*operation phase” (see Appendix Table A1). For Jokkmokksliden, the simulation study for the power calculation could not be carried out using the specific estimates of the parameters obtained from the data due to frequent nonconvergence of the estimation procedure in the simulation. However, by treating the estimated parameters from the combined data as the true parameter values for Jokkmokksliden as well, we were able to run a simulation in which the power was found to be <0.5. The low power of the test using data from Jokkmokksliden was not surprising as the estimates were indeed insignificant.

## 4 Discussion

During the 6‐year study period, we found an absolute decline in reindeer pellet group abundance, at the regional scale up to 15 km from the wind farms and at the local scale close to two relatively small wind farms in a forest summer habitat of freely ranging domesticated reindeer. We investigated the changes in the density of the pellet group abundance in relation to distance to the wind farms. The reindeer habitat use, represented by the pellet group counts related to environmental factors in a binomial model, showed no effect of distance to wind farm area during construction at either the regional or nor the local scale. Our analysis did show a significant increase in habitat use with increased distance to the wind farm at the regional scale during the operation phase of the wind farms. However, at the local scale, at Storliden, we found that reindeer habitat use increased in proximity to the wind farm.

Factors that may have affected abundance of pellet groups irrespective of abundance of reindeer and the wind farm activities that we could not control for in our analysis were herding activities, predator presence, fieldwork activity, and insect harassment. As explained earlier, according to the reindeer herders, the herding actions in the area did not vary much over the study period. Increased predator presence in the area, especially from brown bears predating on reindeer calves right after calving (Karlsson et al., 2012), might be an alternative explanation to reindeer reducing their use of these calving grounds. On the other hand, brown bear are well known to avoid human activity and infrastructure (Nellemann et al., 2007). Furthermore, a recent study of reindeer–brown bear interaction shows that the reindeer could not escape predation from brown bear within their calving ranges during the calving season (Sivertsen et al., 2016), suggesting that any annual change in brown bear abundance not would have changed large‐scale habitat selection by reindeer. The fieldwork in itself could have disturbed the reindeer, as we needed to do the counting after snowmelt but before greening up, that is, at the end of the calving season when the reindeer are known to be sensitive to human activity (e.g., Anttonen, Kumpula, & Colpaert, 2011). However, the inventory could only have changed reindeer behavior during a couple of days (Table 1) and with fairly low impact as the field worker operated alone. More importantly, we only have 1 year of pellet group counts before construction started, which could have been an exceptional year when the reindeer used this area more than normal. However, both data from GPS‐marked reindeer (Skarin et al., 2015) and the first year of pellet group counts when cleaning the plots (not included in the analysis) revealed high use and density of pellets the years preceding this study (Lee et al., 2016; Skarin et al., 2013). Furthermore, precipitation may accelerate the decay of the pellets (Skarin, 2008). The precipitation record from Adak indicates that the May–October period in the years before and during construction (range sum of precipitation 430–559 mm, 2009–2011) was wetter than in the years during operation (280–523 mm, 2012–2014). This implies that the abundance of pellet groups may actually have been underestimated at the beginning of the period in relation to the operation phase, which is also confirmed by the large negative effect estimate of the coefficient associated with the precipitation variable (Table 3). Thus, the decline in pellet groups in the area is probably due to fewer reindeer using the whole region, and as far as our habitat use analysis can explain, this might be connected to the development of the two wind farms, although there could be other reasons for this decline in absolute number of pellet groups.

It was surprising that we did not find any effect of the wind farms at any scale during the construction phase, but we did at the regional scale during the operation phase. In contrast, Colman et al. (2013) found avoidance by reindeer during construction but not during operation at the Kjøllefjord wind farm on the Nordkynn peninsula in northern Norway. Walter, Leslie, and Jenks (2006) showed that Rocky Mountain elk was not at all affected by a wind farm development with respect to home range and dietary needs. Similarly, Taylor, Beck, and Huzurbazar (2016) found no effect of higher winter mortality for pronghorn in relation to development of a wind farm including 74 turbines. However, these studies, like our pellet group count, did not record animal movement, as we hypothesize change in habitat use might be the primary effect of such installations. A parallel study in the same area using GPS data to study reindeer migration routes and movement corridors revealed that the reindeer use of the migration route over the spine road in the area, south of Storliden, was sustained throughout the construction phase, while the use of other corridors north of Storliden almost ceased (Skarin et al., 2015). The continued use of this particular migration corridor could explain why we did not find any avoidance during the construction phase (Figure A1). Furthermore, it was found in the GPS study that reindeer formed a “holding pattern” close to the spine road during the construction years, that is, they waited (possibly due to increased numbers of vehicles on the road) on the eastern side of the road until it was empty to cross freely. This behavior could distort any smoothed effect of distance (measures with a smooth function, e.g., square root). In the GPS study, reindeer movement rate (activity) also increased up to 5 km from the wind farms during the construction phase, indicating a negative effect of the wind farm construction that would not be revealed by pellet group counts at either of the two scales. Moreover, it may not be surprising that reindeer differ from other ungulates in response to disturbance (Taylor et al., 2016; Walter et al., 2006): Reindeer is a highly gregarious species and may react to disturbances to a greater extent than elk and pronghorns. More surprising was the difference in results compared to the reindeer study by Colman et al. (2013). However, the Colman et al. (2013) study differed significantly from our study in terms of methodology, as well as with regard to general environmental conditions. Their study was performed at an intermediate scale (cf. Skarin & Åhman, 2014) as the study area was located above the tree line and surrounded by sea on three sides, preventing the reindeer from escaping the area. This limited data sampling and response of reindeer behavior to within 6 km of the wind farm and they compared this sampling with a similar adjacent peninsula used as a control area (Control‐Impact (CI) design). When evaluating environmental impact on habitat selection by large herbivores, such as reindeer, it is usually problematic to find good control areas as reindeer move across large tracts of land, and we do not want to miss the impact at the regional scale (Northrup et al., 2016; Skarin & Åhman, 2014). Thus, this study could not estimate avoidance at the larger spatial scale (i.e., further away than 4–5 km) that is clearly the most commonly observed impact of industrial development on reindeer and caribou. The fact that the odds ratio of pellet group presence varied according to the distance from the turbines during the construction years (2011 and 2012) in our study could be explained by the human activities not occurring the whole time during this period. For example, construction activity was paused in July each year due to workers’ vacations, whereas the wind turbines in operation run throughout the year rotating and making a sound both day and night, although the human activity is less and the sudden sounds that can happen during construction work are absent.

The result during the operation phase at the local scale at Storliden of increased use closer to the wind farm might be explained by the fact that the reindeer simply did not find the wind farm in operation disturbing (Flydal, Eftestøl, Reimers, & Colman, 2004). However, this does not explain our finding of avoidance of the wind farm at the regional scale and the decrease in absolute pellet group density at Storliden during the operation phase. Like the local scale study by Flydal et al. (2004) and the study at the intermediate scale by Colman et al. (2013), we could not expect to discover the whole reindeer behavioral response within the local scales in our study as this excludes behavioral decisions of the animal at larger geographical scales (Northrup et al., 2016; Skarin & Åhman, 2014). Our pellet group count covered reindeer use during the whole snow‐free season including the insect harassment period in mid‐summer. The wind farm area with roads and open habitat high up in the terrain might provide good insect relief. It is well‐known that both reindeer and caribou show higher tolerance toward human activity or infrastructure to avoid insect harassment (Pollard, Ballard, Noel, & Cronin, 1996; Skarin, Danell, Bergstrom, & Moen, 2004). This, in combination with a possible continued and more concentrated use of the southern migration route also during the operation phase could explain increased use closer to the wind farm. More detailed studies of reindeer behavior during the operation phase could reveal the mechanisms behind these contradictory results both between the local and regional scales and between construction and operation phases.

The analysis of the combined data and the regional data showed that clear cut was the most preferred vegetation type in this region, which may be explained by the high nutritional value and lower density of predators (Dussault et al., 2012; Sivertsen et al., 2016). Skarin et al. (2015), analyzing habitat selection in the same area, reported that clear cuts were also the most important vegetation type. For forest‐dwelling domesticated reindeer, these open habitats might also be important later in the summer season in relation to insect harassment (Helle, Aspi, Lempa, & Taskinen, 1992). This preference for clear cuts could also explain the high absolute abundance of pellet groups at Storliden and low abundance at Jokkmokksliden. Storliden had a higher density of clear cuts and was also partly surrounded by open mires, compared to Jokkmokksliden, which mainly consisted of young (dense) forest planted with *Pinus contorta* known to be avoided by reindeer (Kumpula, Colpaert, & Anttonen, 2007). At Jokkmokksliden, the abundance of counted pellet groups was low from the start and there was only a nonsignificant decline in pellets in relation to the distance to the wind farm. The fact that Jokkmokksliden was avoided by reindeer was also confirmed by the reindeer herders, as reported in previous publications (Skarin et al., 2013, 2015).

In conclusion, we discovered a preference for the wind farm area at Storliden at the local scale and we could not reject the null hypothesis that wind farms do not affect reindeer habitat use at this scale, although the same hypothesis was rejected at the regional scale. Explanations for these contradictory results could be found, although our results could not reveal the mechanisms behind the different effects at the local and the regional scales, respectively. We therefore believe that more detailed information, such as GPS‐data, is needed to explain reindeer habitat use and behavior around wind farms in operation. Thus, even small wind farms with their associated infrastructure may displace freely ranging domesticated reindeer after construction when in operation. Our study also confirms the importance of examining both the regional and local scale. BA design, including the regional scale and taking into account other habitat variability, could therefore be recommended in preference to BACI or CI designs that only consider the local scale, in environmental studies when examining a migratory animal moving over large areas, so as to not miss important information.

## Conflict of Interest

None declared.

## Appendix 2

### Power Calculation

The Monte Carlo simulation for the power calculation was conducted in the following way:

1. Take whole data and subsets of the whole data representing, Jokkmokksliden, Storliden, and the region.
2. Fit a binary hglm (same as those in Table 3) to them with a CAR random effect, using the EQL method, to obtain an estimate for the parameter value. This gives essentially the same parameter estimates as those given in Table 3.
3. Simulate the spatial random effects from a Gaussian distribution with zero mean and estimated covariance from 2. However, for Jokkmokksliden, the parameter values are taken from the combined data (See Table 3, first column).
4. Simulate response variable with same covariates, and their effects estimates from 2 and random effects from 3.
5. Refit the model and extract *p*‐values associated with the two interaction terms (distance*phase) and check whether the *p*‐values are less than .05.
6. Repeat 3–5 a total of 1,000 times and determine the proportion of runs in which H_0_ is rejected (at *p*‐value < .05); this gives the empirical power of the test.

The results in Table 3 were obtained by using the first order correction to the EQL method (EQL1; see Alam et al., 2015; for detailed description of the methods). However, the simulation study was conducted without using first‐order correction (i.e., method=“EQL”, was used in hglm) because the EQL1 was computationally very slow and it often failed to converge in the simulation study. Because, we know that EQL1 provides better estimates of the parameter than EQL (see Alam et al., 2015), the power of the test for EQL1 will not be lower than that of EQL.

**Table A1:** Power of the test for observations of pellet group in relation to Distance to wind turbine parameters, estimated with the binomial model

Parameter	Area			
	Combined (988)	Region (812)	Storliden (920)	Jokkmokksliden^a^ (952)
Sqrt (Dist. to wind turbine/100 m)	0.58	0.17	0.47	0.10
Sqrt (Dist to wind turbine/100)^a^ *Construction phase	0.53	0.51	0.59	0.44
Sqrt (Dist to wind turbin/100)^a^ *Operation phase	0.74	0.63	0.82	0.34

Values in parentheses indicate the number of convergent iterations of 1,000.

a Parameter values are taken from the “Combined” data analysis because the simulation study was unsuccessful with the original parameter estimates obtained from the original Jokkmokksliden data.
